# Supplementary material for: Variation in methods, results and reporting in electronic health record-based studies evaluating routine care in gout: A systematic review
Source: PLoS One. 2019 Oct 24;14(10):e0224272. doi: 10.1371/journal.pone.0224272 (PMC6812805; doi:10.1371/journal.pone.0224272)
Supplement: S1 Fig — The dotted line represents a polynomial regression line. (PDF) [file pone.0224272.s001.pdf]

Supplementary Figure 1. Frequency of articles by publication year (n = 74)

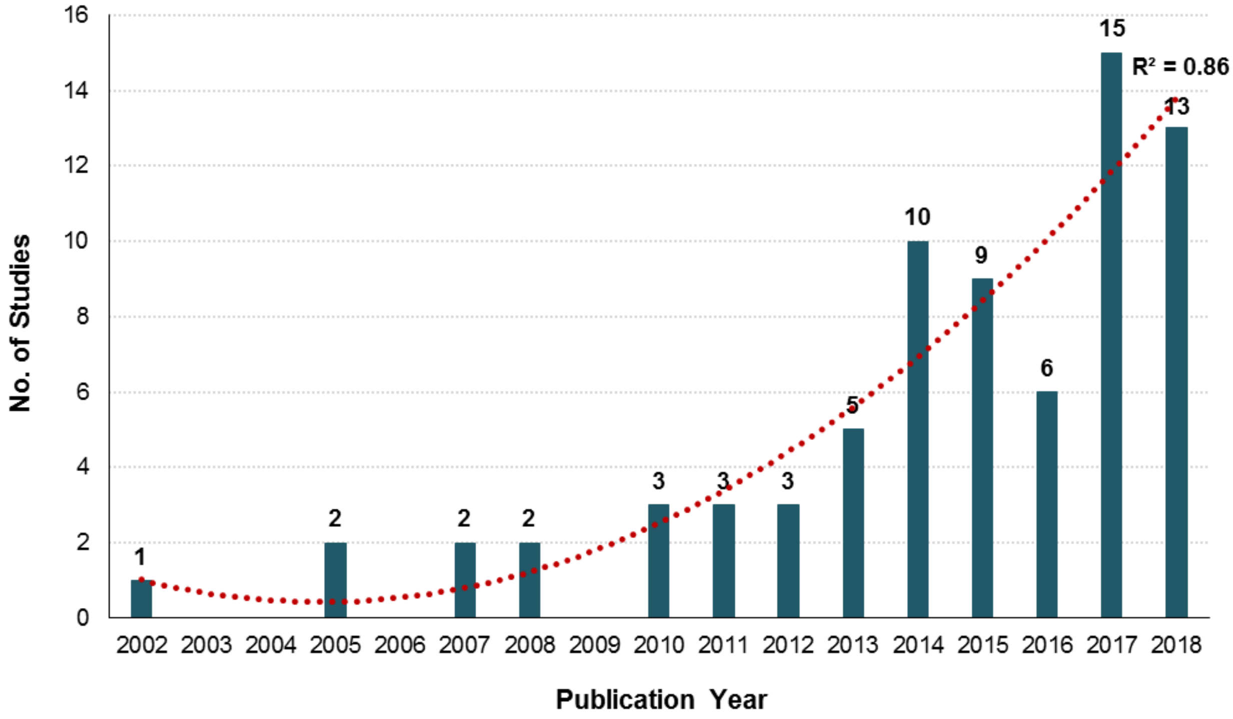

Note: The dotted line represents a polynomial regression line
