# Supplementary material for: Variation in methods, results and reporting in electronic health record-based studies evaluating routine care in gout: A systematic review
Source: PLoS One. 2019 Oct 24;14(10):e0224272. doi: 10.1371/journal.pone.0224272 (PMC6812805; doi:10.1371/journal.pone.0224272)
Supplement: S2 Fig — Horizontal lines are medians and interquartile ranges (25th and 75th percentiles); whiskers’ ends indicate the maximum and minimum values at most 1.5 times the interquartile range from the hinge; dark individual dots are outlier values. (PDF) [file pone.0224272.s002.pdf]

Supplementary Figure 2. Boxplot of overall CoR scores for studies by publication year (n = 74)

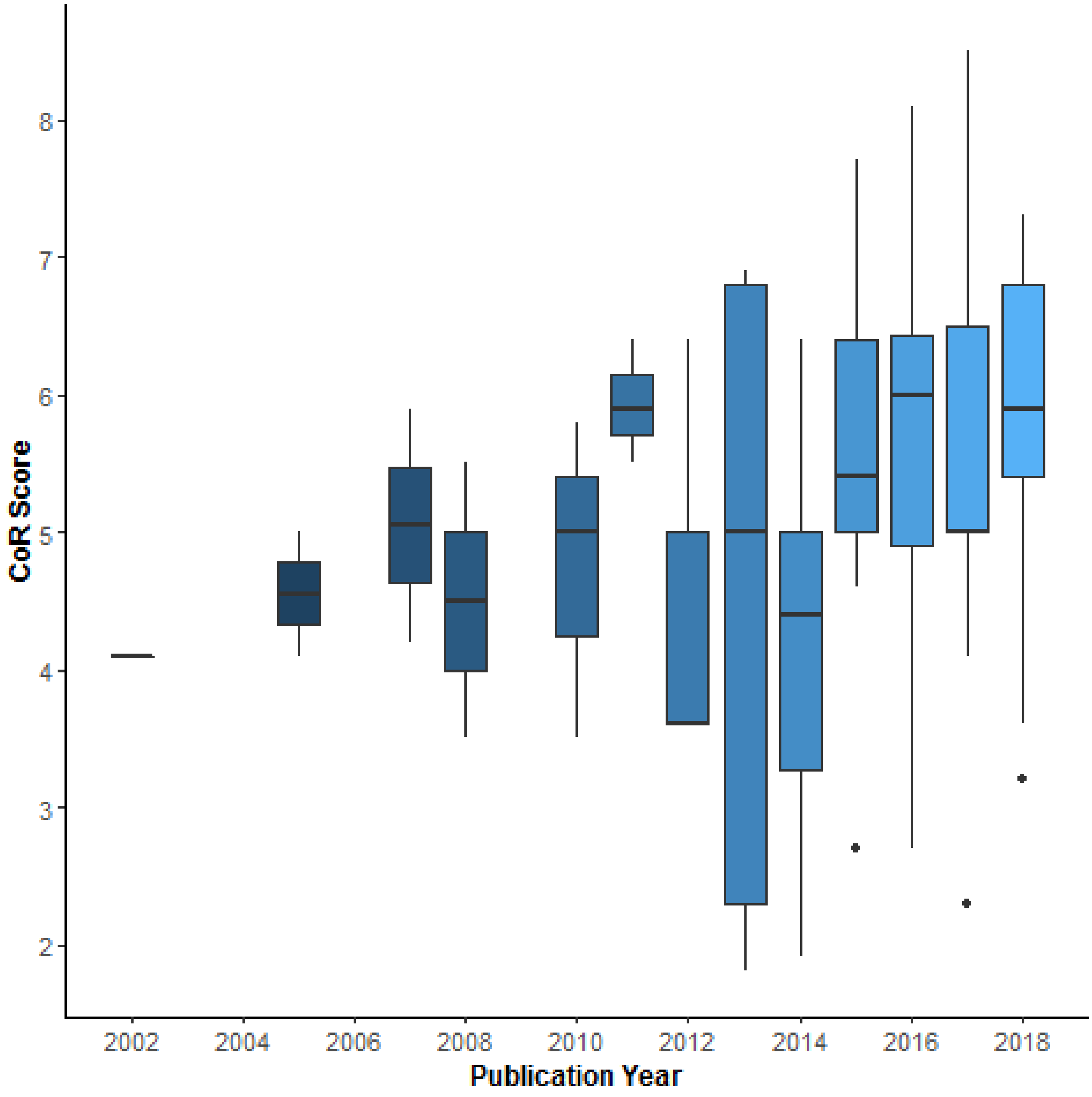

Note: Horizontal lines are medians and interquartile ranges (25th and 75th percentiles); whiskers' ends indicate the maximum and minimum values at most 1.5 times the interquartile range from the hinge; dark individual dots are outlier values.
