# Supplementary material for: Variation in methods, results and reporting in electronic health record-based studies evaluating routine care in gout: A systematic review
Source: PLoS One. 2019 Oct 24;14(10):e0224272. doi: 10.1371/journal.pone.0224272 (PMC6812805; doi:10.1371/journal.pone.0224272)
Supplement: S4 Fig — The blue line is the smooth local weighted regression line (LOESS curve). The shaded area indicates the 95% confidence interval. (PDF) [file pone.0224272.s004.pdf]

Supplementary Figure 4. Scatterplot of overall RoB scores for studies by cohort size (n = 75)

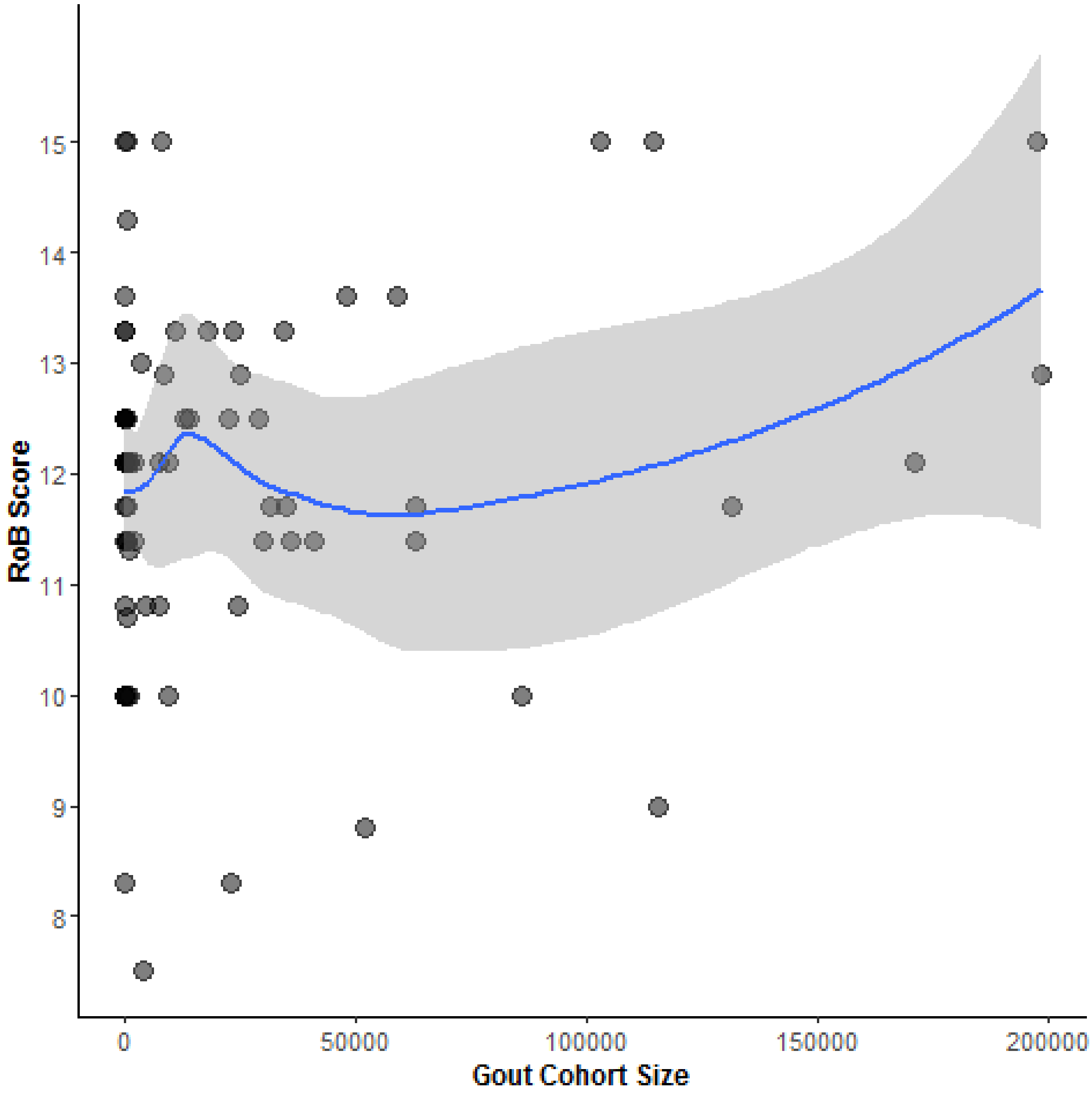

Note: The blue line is the smooth local weighted regression line (LOESS curve). The shaded area indicates the 95% confidence interval
