# Supplementary material for: Variation in methods, results and reporting in electronic health record-based studies evaluating routine care in gout: A systematic review
Source: PLoS One. 2019 Oct 24;14(10):e0224272. doi: 10.1371/journal.pone.0224272 (PMC6812805; doi:10.1371/journal.pone.0224272)
Supplement: S2 Table — MeSH terms are indicated by ‘+’ and a wildcard by ‘*’. (PDF) [file pone.0224272.s006.pdf]

Supplementary Table 2. Search terms used with synonyms.

EHR	Gout	Medication
Electronic health record+	Podagra	Treatment
Medical records systems+	Gouty	Pharmacotherapy
Record-linkage	Arthritis, gouty+	Drug*
Routin* ADJ5 data		Allopurinol+
(Electronic OR link* OR compute* OR anonymi*ed) ADJ5 record		Benzbromarone+
(Health OR patient OR clinic* OR medic* OR care) AND (record* OR data OR plan* OR chart*) AND (compute* OR system OR electronic OR warehouse OR link* OR dataset OR network)		Medication systems+
		Drug therapy+
“System”		Proben*cid+
EPR		Sulfinpyrazone+
EMR		Sulphinpyrazone
EHR		Colchicine+
Database		Febuxostat+
Datalink		“Xanthine oxidase”+
		“Urate lowering”
		ULT
		Prescribing
		Therapy
		“Anti-rheumatic drug”
		Drug prescriptions+
		“gout suppressant”*+
		“anti-gout agent”*
		Prescriptions+
		Non-steroidal anti-inflammator*

		NSAID<br>Anti-Inflammatory Agents, Non-Steroidal+<br>Uricosuric<br>Uricorsuric agent*+<br>Medication therapy management+<br>Drug therapy management+<br>“drug monitoring”+<br>Pharmacovigilance+<br>“pharmaceutical preparations”+<br>Prescription drugs+<br>Drugs, generic+<br>Prescription<br>“antirheumatic agent”*+<br>Medication adherence+<br>Adheren*

MeSH terms are indicated by ‘+’ and a wildcard by ‘*’
