# Supplementary material for: Variation in methods, results and reporting in electronic health record-based studies evaluating routine care in gout: A systematic review
Source: PLoS One. 2019 Oct 24;14(10):e0224272. doi: 10.1371/journal.pone.0224272 (PMC6812805; doi:10.1371/journal.pone.0224272)
Supplement: S3 Table — References are cited in S1 File. (PDF) [file pone.0224272.s007.pdf]

Supplementary Table 3. Aim of studies included in the review (n = 75)

Author (Reference)	Title	Aim
Harrold et al. (4)	The dynamics of chronic gout treatment: Medication gaps and return to therapy	Adherence and gaps in therapy
Dehlin et al. (5)	Factors associated with initiation and persistence of urate-lowering therapy	Adherence and gaps in therapy
Scheepers et al. (6)	Medication adherence among gout patients initiated allopurinol: a retrospective cohort study in the Clinical Practice Research Datalink (CPRD)	Adherence and gaps in therapy
Zandman-Goddard et al. (7)	Rates of adherence and persistence with allopurinol therapy among gout patients in Israel	Adherence and gaps in therapy
Mikuls et al. (8)	Adherence and outcomes with urate-lowering therapy: a site-randomized trial	Adherence and gaps in therapy
Rashid et al. (9)	Modifiable factors associated with allopurinol adherence and outcomes among patients with gout in an integrated healthcare system	Adherence and gaps in therapy; treatment effectiveness
Mantarro et al. (10)	Allopurinol adherence among patients with gout: an Italian general practice database study	Adherence and gaps in therapy; treatment effectiveness
Coburn et al. (11)	Allopurinol Medication Adherence as a Mediator of Optimal Outcomes in Gout Management	Adherence and gaps in therapy; treatment effectiveness
Singh et al. (12)	Quality of care for gout in the US needs improvement	Adherence to clinical guidelines
Singh et al. (13)	Opportunities for improving medication use and monitoring in gout	Adherence to clinical guidelines
Cottrell et al. (14)	Improvement in the management of gout is vital and overdue: an audit from a UK primary care medical practice	Adherence to clinical guidelines
Hmar et al. (15)	Understanding and improving the use of allopurinol in a teaching hospital	Adherence to clinical guidelines
Hughes et al. (16)	Monitoring of urate-lowering therapy among us veterans following the 2012 American College of Rheumatology Guidelines for Management of Gout	Adherence to clinical guidelines
Hassan and Choudry (17)	The compliance of guidelines set by the British Society for Rheumatology for managing Gout	Adherence to clinical guidelines
Jackson et al. (18)	Variation in gout care in Aotearoa New Zealand: a national analysis of quality markers	Adherence to clinical guidelines
Mikuls et al. (19)	Suboptimal physician adherence to quality indicators for the management of gout and asymptomatic hyperuricaemia: results from the UK General Practice Research Database (GPRD)	Adherence to clinical guidelines
Clarson et al. (20)	Factors influencing allopurinol initiation in primary care	Adherence to clinical guidelines
Kuo et al. (21)	Eligibility for and prescription of urate-lowering treatment in patients with incident gout in England	Adherence to clinical guidelines
George et al. (22)	Evaluating appropriate use of prophylactic colchicine for gout flare prevention	Adherence to clinical guidelines
Kerr et al. (23)	Measuring physician adherence with gout quality indicators: a role for natural language processing	Adherence to clinical guidelines
Kapetanovic et al. (24)	Prevalence and incidence of gout in southern Sweden from the socioeconomic perspective	Epidemiology of gout
Meek et al. (25)	Hyperuricaemia: A marker of increased cardiovascular risk in rheumatic patients: Analysis of the ACT-CVD cohort	Epidemiology of gout
Alonso et al. (26)	Gout and risk of Parkinson disease: a prospective study	Epidemiology of gout

Sultan et al. (27)	Gout and subsequent erectile dysfunction: a population- based cohort study from England	Epidemiology of gout
Bevis et al. (28)	Comorbidity clusters in people with gout: an observational cohort study with linked medical record review	Epidemiology of gout
Fisher et al. (29)	The unclosing premature mortality gap in gout: a general population-based study	Epidemiology of gout
MacFarlane et al. (30)	The effect of initiating pharmacologic insulin on serum uric acid levels in patients with diabetes: A matched cohort analysis	Epidemiology of gout
Olaru et al. (31)	Coexistent rheumatoid arthritis and gout: a case series and review of the literature	Epidemiology of gout
Rashid et al. (32)	Patient and clinical characteristics associated with gout flares in an integrated healthcare system	Epidemiology of gout
Sigurdardottir et al. (33)	Work disability in gout: a population-based case-control study	Epidemiology of gout
Chang et al. (34)	Association between gout and aortic stenosis	Epidemiology of gout
DeVera et al. (35)	Gout and the risk of Parkinson's disease: a cohort study	Epidemiology of gout
Landgren et al. (36)	Incidence of and risk factors for nephrolithiasis in patients with gout and the general population, a cohort study	Epidemiology of gout
Spaetgens et al. (37)	Risk of infections in patients with gout: a population-based cohort study	Epidemiology of gout
Lu et al. (38)	Gout and the risk of Alzheimer's disease: a population-based, BMI-matched cohort study	Epidemiology of gout
Rho et al. (39)	Independent impact of gout on the risk of diabetes mellitus among women and men: a population-based, BMI-matched cohort study	Epidemiology of gout
Wahedduddin et al. (40)	Gout in the Hmong in the United States	Epidemiology of gout
Cheyoe et al. (41)	The prevalence of chronic kidney disease among gout patients in Nongjik hospital, Pattani province	Epidemiology of gout
Nyberg et al. (42)	Comorbidity burden in trial-aligned patients with established gout in Germany, UK, US, and France: A retrospective analysis	Epidemiology of gout
Rothenbacher et al. (43)	Frequency and risk factors of gout flares in a large population-based cohort of incident gout	Epidemiology of gout
Arromdee et al. (44)	Epidemiology of gout: is the incidence rising?	Epidemiology of gout
Lee et al. (45)	Elderly patients exhibit stronger inflammatory responses during gout attacks	Epidemiology of gout
Robinson et al. (46)	An observational study of gout prevalence and quality of care in a national australian general practice population	Epidemiology of gout; Adherence to clinical guidelines
Dehlin et al. (47)	Incidence and prevalence of gout in Western Sweden	Epidemiology of gout; Patient management
Rai et al. (48)	The rising prevalence and incidence of gout in British Columbia, Canada: Population-based trends from 2000 to 2012	Epidemiology of gout; Patient management
Kuo et al. (49)	Rising burden of gout in the UK but continuing suboptimal management: a nationwide population study	Epidemiology of gout; Patient management
Soriano et al. (50)	Contemporary epidemiology of gout in the UK general population	Epidemiology of gout; Patient management
Zarowitz and O'Shea (51)	Demographic and clinical profile of nursing facility residents with gout	Epidemiology of gout; Patient management
Mikuls et al. (52)	Gout epidemiology: results from the UK General Practice Research Database, 1990-1999	Epidemiology of gout; Patient management
Maravic et al. (53)	Persistent clinical inertia in gout in 2014: An observational French longitudinal patient database study	Epidemiology of gout; Patient management
Kuo et al. (54)	Urate-lowering treatment and risk of total joint replacement in patients with gout	Epidemiology of gout; Treatment safety
Sultan et al. (55)	Risk of fragility fracture among patients with gout and the effect of urate-lowering therapy	Epidemiology of gout; Treatment safety

Roughley et al. (56)	Risk of chronic kidney disease in patients with gout and the impact of urate lowering therapy: A population-based cohort study	Epidemiology of gout; Treatment safety
Harrold et al. (57)	Patients' knowledge and beliefs concerning gout and its treatment: a population based study	Patient knowledge, beliefs and education
Dehlin and Jacobsson (58)	Trends in gout hospitalization in Sweden	Patient management
Keenan et al. (59)	Prevalence of contraindications and prescription of pharmacologic therapies for gout	Patient management
Morlock et al. (60)	Disease control, health resource use, healthcare costs, and predictors in gout patients in the United States, the United Kingdom, Germany, and France: A retrospective analysis	Patient management
Park et al. (61)	Clinical factors and treatment outcomes associated with failure in the detection of urate crystal in patients with acute gouty arthritis	Patient management
Roddy et al. (62)	Prescription and comorbidity screening following consultation for acute gout in primary care	Patient management
Lin et al. (63)	Cost-effectiveness of an adherence-enhancing intervention for gout based on real-world data	Patient management
Janssen et al. (64)	Quality of care in gout: a clinical audit on treating to the target with urate lowering therapy in real-world gout patients	Treatment effectiveness
Hatoum et al. (65)	Achieving Serum Urate Goal: A comparative effectiveness study between allopurinol and febuxostat	Treatment effectiveness
Jung et al. (66)	Effect of fenofibrate on uric acid level in patients with gout	Treatment effectiveness
Pui et al. (67)	Efficacy and tolerability of probenecid as urate-lowering therapy in gout; clinical experience in high-prevalence population	Treatment effectiveness; Treatment safety
Thueringer et al. (68)	Anakinra for the treatment of acute severe gout in critically ill patients	Treatment effectiveness; Treatment safety
Kuo et al. (69)	Effect of allopurinol on all-cause mortality in adults with incident gout: propensity score-matched landmark analysis	Treatment safety
Dennison et al. (70)	Is allopurinol use associated with an excess risk of osteoporotic fracture? A national prescription registry study	Treatment safety
Solomon et al. (71)	Effects of colchicine on risk of cardiovascular events and mortality among patients with gout: a cohort study using electronic medical records linked with Medicare claims	Treatment safety
Coburn et al. (72)	Allopurinol dose escalation and mortality among patients with gout: a national propensity-matched cohort study	Treatment safety
Keller et al. (73)	Statin use and mortality in gout: A general population-based cohort study	Treatment safety
Ryu et al. (74)	Clinical risk factors for adverse events in allopurinol users	Treatment safety
Crittenden et al. (75)	Colchicine use is associated with decreased prevalence of myocardial infarction in patients with gout	Treatment safety
Kwon et al. (76)	Risk of colchicine-associated myopathy in gout: influence of concomitant use of statin	Treatment safety
Vargas-Santos et al. (77)	Association of chronic kidney disease with allopurinol use in gout treatment	Treatment safety
Lee et al. (78)	Hepatic Safety of Febuxostat Compared with Allopurinol in Gout Patients with Fatty Liver Disease	Treatment safety

References are cited in S1 File.
