# Supplementary material for: Variation in methods, results and reporting in electronic health record-based studies evaluating routine care in gout: A systematic review
Source: PLoS One. 2019 Oct 24;14(10):e0224272. doi: 10.1371/journal.pone.0224272 (PMC6812805; doi:10.1371/journal.pone.0224272)
Supplement: S4 Table — NSAID, non-steroidal anti-inflammatory; ULT, urate lowering therapy. (PDF) [file pone.0224272.s008.pdf]

Supplementary Table 4. Gout medication types in the studies (n = 75)

Medication Type	Count (%)
Urate lowering therapy	72 (96)
Allopurinol	61 (81)
Febuxostat	16 (21)
Oxypurinol	1 (1)
ULT group	21 (28)
Prophylactic	53 (71)
NSAIDs	42 (56)
Colchicine	41 (55)
Corticosteroid	28 (37)
Other analgesic	9 (12)
Prophylactic group	1 (1)
Probenicid	19 (25)
Benzbromarone	5 (7)
Sulfinpyrazone	6 (8)
Uricosuric drugs	2 (3)
Uricosuric antihypertensive / diuretic	4 (5)
Prednisolone	2 (3)
Pegloticase	2 (3)
Other (medications only used in one study)	4 (5)

Note: NSAID, non-steroidal anti-inflammatory; ULT, urate lowering therapy
