# Supplementary material for: Variation in methods, results and reporting in electronic health record-based studies evaluating routine care in gout: A systematic review
Source: PLoS One. 2019 Oct 24;14(10):e0224272. doi: 10.1371/journal.pone.0224272 (PMC6812805; doi:10.1371/journal.pone.0224272)
Supplement: S5 Table — RECORD = REporting of studies Conducted using Observational Routinely-collected Data. References are cited in S1 File. (PDF) [file pone.0224272.s009.pdf]

Supplementary Table 5. Frequency of studies with comprehensive reporting on RECORD items and additional relevant items (n = 75) (79)

RECORD	Question	Count (%)			
		Yes	Partly	No	NA
1.1	Is the type of data used specified in the title or abstract (with database name if applicable)?	**49 (65)**	17 (23)	9 (12)	
1.2	Is the geographic region and timeframe within which the study took place reported in the title or abstract?	**32 (43)**	25 (33)	18 (24)	
1.3	If databases were linked as part of the study, is this stated in the title or abstract?	13 (17)	2 (3)	17 (23)	**43 (57)**
6.1	In the methods, is the method of study cohort selection (e.g. codes or algorithm used to identify subjects) listed in detail, or an explanation as to why this is not shared?	23 (31)	**47 (63)**	5 (7)	
6.2	In the methods, was any validation conducted during study published?	6 (8)	20 (27)	**49 (65)**	
6.3	Is there a flow diagram / graph with the number of individuals in the data at each stage (e.g. of linkage or cohort selection)?	25 (33)	5 (7)	**45 (60)**	
7.1	Are codes or algorithms provided for all exposures, outcomes, confounders and effect modifiers, or an explanation as to why this is not shared?	7 (9)	**53 (71)**	15 (20)	
12.1	Do authors describe the extent to which they had access to the database population used to create the study creation?	**51 (68)**	17 (23)	7 (9)	
12.2	Is information provided on the data cleaning methods?	4 (5)	27 (36)	**44 (59)**	
12.3	If databases were linked as part of the study, are methods of linkage and linkage quality evaluation provided, and is linkage at the person, organization or other level?	7 (9)	14 (19)	11 (15)	**43 (57)**
13.1	In the results, is the cohort selection described in detail, including filtering based on data quality, data availability and linkage? (text or diagram)	27 (36)	**46 (61)**	2 (3)	
19.1	In the discussion, is there discussion about the implications of using data not primarily collected for the study? For example, discussion of misclassification bias, unmeasured confounding, changing eligibility over time	**55 (73)**	16 (21)	4 (5)	
22.1	Authors provide information on how to access any supplemental information such as the study protocol, raw data, or programming code	6 (8)	22 (29)	**47 (63)**	
NA	Study observation period, start and end dates (month, year)	**47 (63)**	25 (33)	3 (4)	
NA	Count of sites contributing to the source database	**39 (52)**		36 (48)	
NA	Count of patients in the source database	31 (41)		**44 (59)**	

Note: RECORD = REporting of studies Conducted using Observational Routinely-collected Data. References are cited in S1 File.
