# Supplementary material for: Variation in methods, results and reporting in electronic health record-based studies evaluating routine care in gout: A systematic review
Source: PLoS One. 2019 Oct 24;14(10):e0224272. doi: 10.1371/journal.pone.0224272 (PMC6812805; doi:10.1371/journal.pone.0224272)
Supplement: S1 File — (DOC) [file pone.0224272.s010.doc]

# Variation in Methods, Results and Reporting in Electronic Health Record-based Studies Evaluating Routine Care in Gout: A Systematic Review

# Supplementary Information

Samantha S. R. Crossfield, Lana Yin Hui Lai, Sarah R. Kingsbury, Paul Baxter, Owen Johnson, Philip G. Conaghan, Mar Pujades-Rodriguez

Table of Contents

[Supplementary Methods 2](#__RefHeading___Toc15998772)

[Supplementary Tables 4](#__RefHeading___Toc15998773)

[Supplementary Table 1. Preferred Reporting Items for Systematic Reviews and Meta-Analyses (PRISMA) checklist with reference to this review 4](#__RefHeading___Toc15998774)

[Supplementary Table 2. Search terms used with synonyms 6](#__RefHeading___Toc15998775)

[Supplementary Table 3. Aim of studies included in the review 8](#__RefHeading___Toc15998776)

[Supplementary Table 4. Gout medication types in the studies 11](#__RefHeading___Toc15998777)

[Supplementary Table 5. Frequency of studies with comprehensive reporting on RECORD items and additional relevant items 12](#__RefHeading___Toc15998778)

[Supplementary Figures 13](#__RefHeading___Toc15998779)

[Supplementary Figure 1. Frequency of articles by publication year 13](#__RefHeading___Toc15998780)

[Supplementary Figure 2. Boxplot of overall CoR scores for studies by publication year 14](#__RefHeading___Toc15998781)

[Supplementary Figure 3. Boxplot of overall RoB scores for studies by publication year 15](#__RefHeading___Toc15998782)

[Supplementary Figure 4. Scatterplot of overall RoB scores for studies by cohort size 16](#__RefHeading___Toc15998783)

[References 17](#__RefHeading___Toc15998784)

## Supplementary Methods

**Detail of the database search and the filters applied**

The databases searched on 20.02.2019:

1. Scopus (from 1966)
2. Web of Science Core
3. CINAHL
4. PubMed
5. Ovid MEDLINE Epub Ahead of Print, In-Process and Other Non-Indexed Citations, Ovide MEDLINE Daily and Ovid MEDLINE (from 1946)
6. EMBASE Classic + Embase (from 1947)

Search Terms

The search terms were gout, medication and electronic health records (EHRs). A list of synonyms and MeSH for the search terms was derived with clinical guidance (Supplementary Table 2). Synonyms for a term were combined with ‘OR’ and then terms were combined with ‘AND’ so that the string searched was ((“EHR” OR “electronic health record”…) AND (“Gout” OR “podagra”….) AND (“medication” OR “treatment”…)). MeSH terms were used on PubMed, CINAHL, MEDLINE and EMBASE.

Filters

Filters were applied where available to exclude citations published prior to 1970, of non-English language, or with non-human subjects.

Google Scholar

Given constraints in Google Scholar regarding bulk download and complex search query strings, Harzing’s Publish or Perish application (version 6) was used to search the terms (“electronic” AND “record” AND “gout”) AND (“medical” OR “health” OR “patient” OR “clinical” OR “medication” OR “prescription” OR “drug”) and download the first 1000 citations published between 01.01.2000 and 01.08.2017, 01.01.2017 and 07.08.2018, and 08.08.2018 and 20.02.2019 (1).

**Study selection criteria and detail of hand-searching**

Inclusion criteria (all of the following):

1. Study using general population EHR data
2. Selection of prevalent or incident gout patients
3. Reporting on routine gout medication exposure and / or outcomes among gout patients. This included aggregated reporting on medication types, for example, ‘anti-gout medication’

Exclusion criteria (any of the following):

1. EHR data is not used in cohort selection and results (e.g. a case study)
2. Claims, insurance or such databases with no specific reference to data having derived from EHRs (2)
3. EHR-based definitions, data collection and analysis is only reported in combination with data collected from other sources
4. Non-musculoskeletal disease registry or sub-population database, or rare event reporting database
5. Gout is not defined but implied from a definition of hyperuricemia or high serum urate level, which may be asymptomatic, or anti-gout medication, which may be prescribed in other instances such as hematological malignancy
6. Medication management is determined by trial, study-led intervention or simulation
7. Non-human animal study
8. Unavailability of a full manuscript in English despite communication with the main author
9. Publication prior to 1970, the first decade with established EHRs
10. Conference or meeting abstract, research protocol or review. These were excluded from the final cohort

Hand-searching

When a review was identified during screening, which referenced EHR-based studies of gout management, it was included during title and abstract screening and a flag was added during full paper screening. The EHR-related references of flagged reviews were screened and the review itself was excluded.

When a conference, meeting or protocol publication was identified during screening, which was an EHR-based study of gout medication management, it was included during title and abstract screening and a flag was added during full paper screening. For flagged manuscripts, an author search was run for each of the first, second and last authors alongside the term ‘gout’ in Google Scholar to select papers published subsequently for screening. The flagged manuscript was excluded.

## Supplementary Tables

### Supplementary Table 1. Preferred Reporting Items for Systematic Reviews and Meta-Analyses (PRISMA) checklist with reference to this review (3)

| **Section/topic** | **#** | **Checklist item** | **Reported on page #** |
| --- | --- | --- | --- |
| **TITLE** | | |  |
| Title | 1 | Identify the report as a systematic review, meta-analysis, or both. | 1 |
| **ABSTRACT** | | |  |
| Structured summary | 2 | Provide a structured summary including, as applicable: background; objectives; data sources; study eligibility criteria, participants, and interventions; study appraisal and synthesis methods; results; limitations; conclusions and implications of key findings; systematic review registration number. | 2 |
| **INTRODUCTION** | | |  |
| Rationale | 3 | Describe the rationale for the review in the context of what is already known. | 4 |
| Objectives | 4 | Provide an explicit statement of questions being addressed with reference to participants, interventions, comparisons, outcomes, and study design (PICOS). | 4 |
| **METHODS** | | |  |
| Protocol and registration | 5 | Indicate if a review protocol exists, if and where it can be accessed (e.g., Web address), and, if available, provide registration information including registration number. | 4 |
| Eligibility criteria | 6 | Specify study characteristics (e.g., PICOS, length of follow-up) and report characteristics (e.g., years considered, language, publication status) used as criteria for eligibility, giving rationale. | 4 |
| Information sources | 7 | Describe all information sources (e.g., databases with dates of coverage, contact with study authors to identify additional studies) in the search and date last searched. | 4 |
| Search | 8 | Present full electronic search strategy for at least one database, including any limits used, such that it could be repeated. | Supp. Methods |
| Study selection | 9 | State the process for selecting studies (i.e., screening, eligibility, included in systematic review, and, if applicable, included in the meta-analysis). | 4 |
| Data collection process | 10 | Describe method of data extraction from reports (e.g., piloted forms, independently, in duplicate) and any processes for obtaining and confirming data from investigators. | 5 |
| Data items | 11 | List and define all variables for which data were sought (e.g., PICOS, funding sources) and any assumptions and simplifications made. | 5 |
| Risk of bias in individual studies | 12 | Describe methods used for assessing risk of bias of individual studies (including specification of whether this was done at the study or outcome level), and how this information is to be used in any data synthesis. | 5 |
| Summary measures | 13 | State the principal summary measures (e.g., risk ratio, difference in means). | 5 |
| Synthesis of results | 14 | Describe the methods of handling data and combining results of studies, if done, including measures of consistency (e.g., I^2^) for each meta-analysis. | 5 |
| Risk of bias across studies | 15 | Specify any assessment of risk of bias that may affect the cumulative evidence (e.g., publication bias, selective reporting within studies). | NA |
| Additional analyses | 16 | Describe methods of additional analyses (e.g., sensitivity or subgroup analyses, meta-regression), if done, indicating which were pre-specified. | 5 |
| **RESULTS** | | |  |
| Study selection | 17 | Give numbers of studies screened, assessed for eligibility, and included in the review, with reasons for exclusions at each stage, ideally with a flow diagram. | 5 |
| Study characteristics | 18 | For each study, present characteristics for which data were extracted (e.g., study size, PICOS, follow-up period) and provide the citations. | 5-6 |
| Risk of bias within studies | 19 | Present data on risk of bias of each study and, if available, any outcome level assessment (see item 12). | 8-9 |
| Results of individual studies | 20 | For all outcomes considered (benefits or harms), present, for each study: (a) simple summary data for each intervention group (b) effect estimates and confidence intervals, ideally with a forest plot. | NA |
| Synthesis of results | 21 | Present the main results of the review. If meta-analyses are done, include for each, confidence intervals and measures of consistency | 6-9 |
| Risk of bias across studies | 22 | Present results of any assessment of risk of bias across studies (see Item 15). | NA |
| Additional analysis | 23 | Give results of additional analyses, if done (e.g., sensitivity or subgroup analyses, meta-regression [see Item 16]). | 8-9 |
| **DISCUSSION** | | |  |
| Summary of evidence | 24 | Summarize the main findings including the strength of evidence for each main outcome; consider their relevance to key groups (e.g., healthcare providers, users, and policy makers). | 5-8 |
| Limitations | 25 | Discuss limitations at study and outcome level (e.g., risk of bias), and at review-level (e.g., incomplete retrieval of identified research, reporting bias). | 9 |
| Conclusions | 26 | Provide a general interpretation of the results in the context of other evidence, and implications for future research. | 9 |
| FUNDING | | |  |
| Funding | 27 | Describe sources of funding for the systematic review and other support (e.g., supply of data); role of funders for the systematic review. | 1 |

### Supplementary Table 2. Search terms used with synonyms. MeSH terms are indicated by ‘+’ and a wildcard by ‘*’

| **EHR** | **Gout** | **Medication** |
| --- | --- | --- |
| Electronic health record+ | Podagra | Treatment |
| Medical records systems+ | Gouty | Pharmacotherapy |
| Record-linkage | Arthritis, gouty+ | Drug* |
| Routin* ADJ5 data |  | Allopurinol+ |
| (Electronic OR link* OR compute* OR anonymi*ed) ADJ5 record  (Health OR patient OR clinic* OR medic* OR care) AND (record* OR data OR plan* OR chart*) AND (compute* OR system OR electronic OR warehouse OR link* OR dataset OR network) |  | Benzbromarone+ |
|  |  | Medication systems+ |
|  |  | Drug therapy+ |
| “System” |  | Proben*cid+ |
| EPR |  | Sulfinpyrazone+ |
| EMR |  | Sulphinpyrazone |
| EHR |  | Colchicine+ |
| Database |  | Febuxostat+ |
| Datalink |  | “Xanthine oxidase”+ |
|  |  | “Urate lowering” |
|  |  | ULT |
|  |  | Prescribing |
|  |  | Therapy |
|  |  | “Anti-rheumatic drug” |
|  |  | Drug prescriptions+ |
|  |  | “gout suppressant”*+ |
|  |  | “anti-gout agent”* |
|  |  | Prescriptions+ |
|  |  | Non-steroidal anti-inflammator* |
|  |  | NSAID |
|  |  | Anti-Inflammatory Agents, Non-Steroidal+ |
|  |  | Uricosuric |
|  |  | Uricorsuric agent*+ |
|  |  | Medication therapy management+ |
|  |  | Drug therapy management+ |
|  |  | “drug monitoring”+ |
|  |  | Pharmacovigilance+ |
|  |  | “pharmaceutical preparations”+ |
|  |  | Prescription drugs+ |
|  |  | Drugs, generic+ |
|  |  | Prescription |
|  |  | “antirheumatic agent”*+ |
|  |  | Medication adherence+ |
|  |  | Adheren* |

### Supplementary Table 3. Aim of studies included in the review (n = 75)

| **Author (Reference)** | **Title** | **Aim** |
| --- | --- | --- |
| Harrold et al. (4) | The dynamics of chronic gout treatment: Medication gaps and return to therapy | Adherence and gaps in therapy |
| Dehlin et al. (5) | Factors associated with initiation and persistence of urate-lowering therapy | Adherence and gaps in therapy |
| Scheepers et al. (6) | Medication adherence among gout patients initiated allopurinol: a retrospective cohort study in the Clinical Practice Research Datalink (CPRD) | Adherence and gaps in therapy |
| Zandman-Goddard et al. (7) | Rates of adherence and persistence with allopurinol therapy among gout patients in Israel | Adherence and gaps in therapy |
| Mikuls et al. (8) | Adherence and outcomes with urate-lowering therapy: a site-randomized trial | Adherence and gaps in therapy |
| Rashid et al. (9) | Modifiable factors associated with allopurinol adherence and outcomes among patients with gout in an integrated healthcare system | Adherence and gaps in therapy; treatment effectiveness |
| Mantarro et al. (10) | Allopurinol adherence among patients with gout: an Italian general practice database study | Adherence and gaps in therapy; treatment effectiveness |
| Coburn et al. (11) | Allopurinol Medication Adherence as a Mediator of Optimal Outcomes in Gout Management | Adherence and gaps in therapy; treatment effectiveness |
| Singh et al. (12) | Quality of care for gout in the US needs improvement | Adherence to clinical guidelines |
| Singh et al. (13) | Opportunities for improving medication use and monitoring in gout | Adherence to clinical guidelines |
| Cottrell et al. (14) | Improvement in the management of gout is vital and overdue: an audit from a UK primary care medical practice | Adherence to clinical guidelines |
| Hmar et al. (15) | Understanding and improving the use of allopurinol in a teaching hospital | Adherence to clinical guidelines |
| Hughes et al. (16) | Monitoring of urate-lowering therapy among us veterans following the 2012 American College of Rheumatology Guidelines for Management of Gout | Adherence to clinical guidelines |
| Hassan and Choudry (17) | The compliance of guidelines set by the British Society for Rheumatology for managing Gout | Adherence to clinical guidelines |
| Jackson et al. (18) | Variation in gout care in Aotearoa New Zealand: a national analysis of quality markers | Adherence to clinical guidelines |
| Mikuls et al. (19) | Suboptimal physician adherence to quality indicators for the management of gout and asymptomatic hyperuricaemia: results from the UK General Practice Research Database (GPRD) | Adherence to clinical guidelines |
| Clarson et al. (20) | Factors influencing allopurinol initiation in primary care | Adherence to clinical guidelines |
| Kuo et al. (21) | Eligibility for and prescription of urate-lowering treatment in patients with incident gout in England | Adherence to clinical guidelines |
| George et al. (22) | Evaluating appropriate use of prophylactic colchicine for gout flare prevention | Adherence to clinical guidelines |
| Kerr et al. (23) | Measuring physician adherence with gout quality indicators: a role for natural language processing | Adherence to clinical guidelines |
| Kapetanovic et al. (24) | Prevalence and incidence of gout in southern Sweden from the socioeconomic perspective | Epidemiology of gout |
| Meek et al. (25) | Hyperuricaemia: A marker of increased cardiovascular risk in rheumatic patients: Analysis of the ACT-CVD cohort | Epidemiology of gout |
| Alonso et al. (26) | Gout and risk of Parkinson disease: a prospective study | Epidemiology of gout |
| Sultan et al. (27) | Gout and subsequent erectile dysfunction: a population- based cohort study from England | Epidemiology of gout |
| Bevis et al. (28) | Comorbidity clusters in people with gout: an observational cohort study with linked medical record review | Epidemiology of gout |
| Fisher et al. (29) | The unclosing premature mortality gap in gout: a general population-based study | Epidemiology of gout |
| MacFarlane et al. (30) | The effect of initiating pharmacologic insulin on serum uric acid levels in patients with diabetes: A matched cohort analysis | Epidemiology of gout |
| Olaru et al. (31) | Coexistent rheumatoid arthritis and gout: a case series and review of the literature | Epidemiology of gout |
| Rashid et al. (32) | Patient and clinical characteristics associated with gout flares in an integrated healthcare system | Epidemiology of gout |
| Sigurdardottir et al. (33) | Work disability in gout: a population-based case-control study | Epidemiology of gout |
| Chang et al. (34) | Association between gout and aortic stenosis | Epidemiology of gout |
| DeVera et al. (35) | Gout and the risk of Parkinson's disease: a cohort study | Epidemiology of gout |
| Landgren et al. (36) | Incidence of and risk factors for nephrolithiasis in patients with gout and the general population, a cohort study | Epidemiology of gout |
| Spaetgens et al. (37) | Risk of infections in patients with gout: a population-based cohort study | Epidemiology of gout |
| Lu et al. (38) | Gout and the risk of Alzheimer's disease: a population-based, BMI-matched cohort study | Epidemiology of gout |
| Rho et al. (39) | Independent impact of gout on the risk of diabetes mellitus among women and men: a population-based, BMI-matched cohort study | Epidemiology of gout |
| Wahedduddin et al. (40) | Gout in the Hmong in the United States | Epidemiology of gout |
| Cheyoe et al. (41) | The prevalence of chronic kidney disease among gout patients in Nongjik hospital, Pattani province | Epidemiology of gout |
| Nyberg et al. (42) | Comorbidity burden in trial-aligned patients with established gout in Germany, UK, US, and France: A retrospective analysis | Epidemiology of gout |
| Rothenbacher et al. (43) | Frequency and risk factors of gout flares in a large population-based cohort of incident gout | Epidemiology of gout |
| Arromdee et al. (44) | Epidemiology of gout: is the incidence rising? | Epidemiology of gout |
| Lee et al. (45) | Elderly patients exhibit stronger inflammatory responses during gout attacks | Epidemiology of gout |
| Robinson et al. (46) | An observational study of gout prevalence and quality of care in a national australian general practice population | Epidemiology of gout; Adherence to clinical guidelines |
| Dehlin et al. (47) | Incidence and prevalence of gout in Western Sweden | Epidemiology of gout; Patient management |
| Rai et al. (48) | The rising prevalence and incidence of gout in British Columbia, Canada: Population-based trends from 2000 to 2012 | Epidemiology of gout; Patient management |
| Kuo et al. (49) | Rising burden of gout in the UK but continuing suboptimal management: a nationwide population study | Epidemiology of gout; Patient management |
| Soriano et al. (50) | Contemporary epidemiology of gout in the UK general population | Epidemiology of gout; Patient management |
| Zarowitz and O'Shea (51) | Demographic and clinical profile of nursing facility residents with gout | Epidemiology of gout; Patient management |
| Mikuls et al. (52) | Gout epidemiology: results from the UK General Practice Research Database, 1990-1999 | Epidemiology of gout; Patient management |
| Maravic et al. (53) | Persistent clinical inertia in gout in 2014: An observational French longitudinal patient database study | Epidemiology of gout; Patient management |
| Kuo et al. (54) | Urate-lowering treatment and risk of total joint replacement in patients with gout | Epidemiology of gout; Treatment safety |
| Sultan et al. (55) | Risk of fragility fracture among patients with gout and the effect of urate-lowering therapy | Epidemiology of gout; Treatment safety |
| Roughley et al. (56) | Risk of chronic kidney disease in patients with gout and the impact of urate lowering therapy: A population-based cohort study | Epidemiology of gout; Treatment safety |
| Harrold et al. (57) | Patients' knowledge and beliefs concerning gout and its treatment: a population based study | Patient knowledge, beliefs and education |
| Dehlin and Jacobsson (58) | Trends in gout hospitalization in Sweden | Patient management |
| Keenan et al. (59) | Prevalence of contraindications and prescription of pharmacologic therapies for gout | Patient management |
| Morlock et al. (60) | Disease control, health resource use, healthcare costs, and predictors in gout patients in the United States, the United Kingdom, Germany, and France: A retrospective analysis | Patient management |
| Park et al. (61) | Clinical factors and treatment outcomes associated with failure in the detection of urate crystal in patients with acute gouty arthritis | Patient management |
| Roddy et al. (62) | Prescription and comorbidity screening following consultation for acute gout in primary care | Patient management |
| Lin et al. (63) | Cost‐effectiveness of an adherence‐enhancing intervention for gout based on real‐world data | Patient management |
| Janssen et al. (64) | Quality of care in gout: a clinical audit on treating to the target with urate lowering therapy in real-world gout patients | Treatment effectiveness |
| Hatoum et al. (65) | Achieving Serum Urate Goal: A comparative effectiveness study between allopurinol and febuxostat | Treatment effectiveness |
| Jung et al. (66) | Effect of fenofibrate on uric acid level in patients with gout | Treatment effectiveness |
| Pui et al. (67) | Efficacy and tolerability of probenecid as urate-lowering therapy in gout; clinical experience in high-prevalence population | Treatment effectiveness; Treatment safety |
| Thueringer et al. (68) | Anakinra for the treatment of acute severe gout in critically ill patients | Treatment effectiveness; Treatment safety |
| Kuo et al. (69) | Effect of allopurinol on all-cause mortality in adults with incident gout: propensity score-matched landmark analysis | Treatment safety |
| Dennison et al. (70) | Is allopurinol use associated with an excess risk of osteoporotic fracture? A national prescription registry study | Treatment safety |
| Solomon et al. (71) | Effects of colchicine on risk of cardiovascular events and mortality among patients with gout: a cohort study using electronic medical records linked with Medicare claims | Treatment safety |
| Coburn et al. (72) | Allopurinol dose escalation and mortality among patients with gout: a national propensity-matched cohort study | Treatment safety |
| Keller et al. (73) | Statin use and mortality in gout: A general population-based cohort study | Treatment safety |
| Ryu et al. (74) | Clinical risk factors for adverse events in allopurinol users | Treatment safety |
| Crittenden et al. (75) | Colchicine use is associated with decreased prevalence of myocardial infarction in patients with gout | Treatment safety |
| Kwon et al. (76) | Risk of colchicine-associated myopathy in gout: influence of concomitant use of statin | Treatment safety |
| Vargas-Santos et al. (77) | Association of chronic kidney disease with allopurinol use in gout treatment | Treatment safety |
| Lee et al. (78) | Hepatic Safety of Febuxostat Compared with Allopurinol in Gout Patients with Fatty Liver Disease | Treatment safety |

### Supplementary Table 4. Gout medication types in the studies (n = 75)

| **Medication Type** | **Count (%)** |
| --- | --- |
| Urate lowering therapy | 72 (96) |
| Allopurinol | 61 (81) |
| Febuxostat | 16 (21) |
| Oxypurinol | 1 (1) |
| ULT group | 21 (28) |
| Prophylactic | 53 (71) |
| NSAIDs | 42 (56) |
| Colchicine | 41 (55) |
| Corticosteroid | 28 (37) |
| Other analgesic | 9 (12) |
| Prophylactic group | 1 (1) |
| Probenicid | 19 (25) |
| Benzbromarone | 5 (7) |
| Sulfinpyrazone | 6 (8) |
| Uricosuric drugs | 2 (3) |
| Uricosuric antihypertensive / diuretic | 4 (5) |
| Prednisolone | 2 (3) |
| Pegloticase | 2 (3) |
| Other (medications only used in one study) | 4 (5) |

**Note:** NSAID, non-steroidal anti-inflammatory; ULT, urate lowering therapy

### Supplementary Table 5. Frequency of studies with comprehensive reporting on RECORD items and additional relevant items (n = 75) (79)

| **RECORD** | **Question** | **Count (%)** | | | |
| --- | --- | --- | --- | --- | --- |
|  |  | Yes | Partly | No | NA |
| 1.1 | Is the type of data used specified in the title or abstract (with database name if applicable)? | **49**  **(65)** | 17  (23) | 9 (12) |  |
| 1.2 | Is the geographic region and timeframe within which the study took place reported in the title or abstract? | **32**  **(43)** | 25  (33) | 18 (24) |  |
| 1.3 | If databases were linked as part of the study, is this stated in the title or abstract? | 13  (17) | 2  (3) | 17 (23) | **43**  **(57)** |
| 6.1 | In the methods, is the method of study cohort selection (e.g. codes or algorithm used to identify subjects) listed in detail, or an explanation as to why this is not shared? | 23  (31) | **47**  **(63)** | 5 (7) |  |
| 6.2 | In the methods, was any validation conducted during study published? | 6  (8) | 20  (27) | **49 (65)** |  |
| 6.3 | Is there a flow diagram / graph with the number of individuals in the data at each stage (e.g. of linkage or cohort selection)? | 25  (33) | 5  (7) | **45 (60)** |  |
| 7.1 | Are codes or algorithms provided for all exposures, outcomes, confounders and effect modifiers, or an explanation as to why this is not shared? | 7  (9) | **53**  **(71)** | 15 (20) |  |
| 12.1 | Do authors describe the extent to which they had access to the database population used to create the study creation? | **51**  **(68)** | 17  (23) | 7 (9) |  |
| 12.2 | Is information provided on the data cleaning methods? | 4  (5) | 27  (36) | **44 (59)** |  |
| 12.3 | If databases were linked as part of the study, are methods of linkage and linkage quality evaluation provided, and is linkage at the person, organization or other level? | 7  (9) | 14  (19) | 11 (15) | **43**  **(57)** |
| 13.1 | In the results, is the cohort selection described in detail, including filtering based on data quality, data availability and linkage? (text or diagram) | 27  (36) | **46**  **(61)** | 2 (3) |  |
| 19.1 | In the discussion, is there discussion about the implications of using data not primarily collected for the study? For example, discussion of misclassification bias, unmeasured confounding, changing eligibility over time | **55**  **(73)** | 16  (21) | 4 (5) |  |
| 22.1 | Authors provide information on how to access any supplemental information such as the study protocol, raw data, or programming code | 6  (8) | 22  (29) | **47 (63)** |  |
| NA | Study observation period, start and end dates (month, year) | **47**  **(63)** | 25  (33) | 3 (4) |  |
| NA | Count of sites contributing to the source database | **39**  **(52)** |  | 36 (48) |  |
| NA | Count of patients in the source database | 31  (41) |  | **44 (59)** |  |

**Note:** RECORD = REporting of studies Conducted using Observational Routinely-collected Data

###

## Supplementary Figures

### Supplementary Figure 1. Frequency of articles by publication year (n = 74)


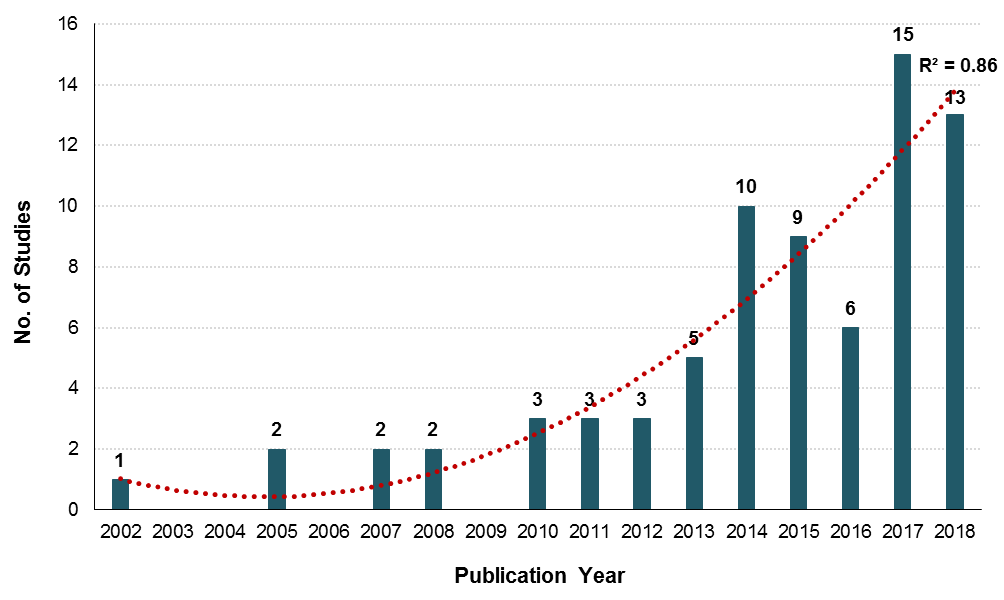


**Note:** The dotted line represents a polynomial regression line

### Supplementary Figure 2. Boxplot of overall CoR scores for studies by publication year (n = 74)


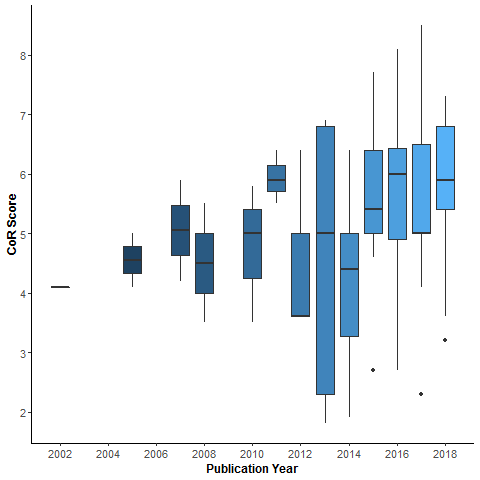


**Note:** Horizontal lines are medians and interquartile ranges (25^th^ and 75^th^ percentiles); whiskers’ ends indicate the maximum and minimum values at most 1.5 times the interquartile range from the hinge; dark individual dots are outlier values.

**Supplementary Figure 3. Boxplot of overall RoB scores for studies by publication year (n = 74)**


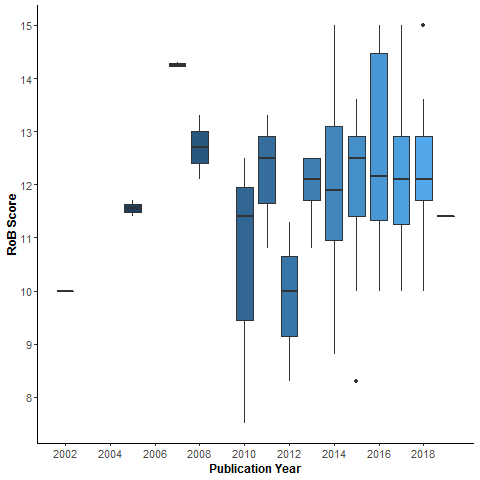


**Note:** Horizontal lines are medians and interquartile ranges (25^th^ and 75^th^ percentiles); whiskers’ ends indicate the maximum and minimum values at most 1.5 times the interquartile range from the hinge; dark individual dots are outlier values.

**Supplementary Figure 4. Scatterplot of overall RoB scores for studies by cohort size (n = 75)**


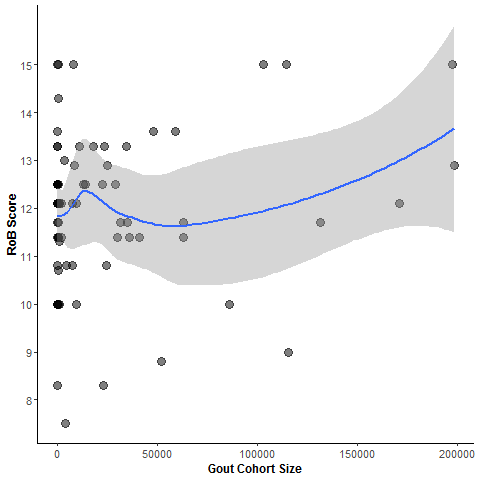


**Note:** The blue line is the smooth local weighted regression line (LOESS curve). The shaded area indicates the 95% confidence interval.

## References

1. Harzing AW. Publish or Perish, Version 5.29.5793. 5.29.5793 ed: Harzing, A. W.; Tarma Software Research; 2017.

2. Schinasi LH, Auchincloss AH, Forrest CB, Diez Roux AV. Using electronic health record data for environmental and place based population health research: a systematic review. Annals of Epidemiology. 2018;28(7):493-502.

3. Moher D, Liberati A, Tetzlaff J, Altman DG, Group P. Preferred reporting items for systematic reviews and meta-analyses: the PRISMA statement. BMJ. 2009;339:b2535.

4. Harrold LR, Andrade SE, Briesacher B, Raebel MA, Fouayzi H, Yood RA, et al. The Dynamics of Chronic Gout Treatment: Medication Gaps and Return to Therapy. Am J Med. 2010;123(1):54-9.

5. Dehlin M, Ekström EH, Petzold M, Strömberg U, Telg G, Jacobsson LTH. Factors associated with initiation and persistence of urate-lowering therapy. Arthritis Res Ther. 2017;19(1):6.

6. Scheepers LEJM, Burden AM, Arts ICW, Spaetgens B, Souverein P, de Vries F, et al. Medication adherence among gout patients initiated allopurinol: a retrospective cohort study in the Clinical Practice Research Datalink (CPRD). Rheumatology (Oxford). 2018;57(9):1641-50.

7. Zandman-Goddard G, Amital H, Shamrayevsky N, Raz R, Shalev V, Chodick G. Rates of adherence and persistence with allopurinol therapy among gout patients in Israel. Rheumatology (Oxford). 2013;52(6):1126-31.

8. Mikuls TR, Cheetham TC, Levy GD, Rashid N. Adherence and Outcomes with Urate-Lowering Therapy: A Site-Randomized Trial. Am J Med. 2019:354-61.

9. Rashid N, Coburn BW, Wu Y-L, Cheetham TC, Curtis JR, Saag KG, et al. Modifiable factors associated with allopurinol adherence and outcomes among patients with gout in an integrated healthcare system. J Rheumatol. 2014;42(3):504-12.

10. Mantarro S, Capogrosso-Sansone A, Tuccori M, Blandizzi C, Montagnani S, Convertino I, et al. Allopurinol adherence among patients with gout: an Italian general practice database study. Int J Clin Pract. 2015;69(7):757-65.

11. Coburn BW, Bendlin KA, Sayles H, Meza J, Russell CL, R MT. Allopurinol Medication Adherence as a Mediator of Optimal Outcomes in Gout Management. J Clin Rheumatol. 2017;23(6):317-23.

12. Singh JA, Hodges JS, Toscano JP, Asch SM. Quality of care for gout in the US needs improvement. Arthritis Rheum. 2007;57(5):822-9.

13. Singh JA, Hodges JS, Asch SM. Opportunities for improving medication use and monitoring in gout. Ann Rheum Dis. 2008;68(8):1265-70.

14. Cottrell E, Crabtree V, Edwards JJ, Roddy E. Improvement in the management of gout is vital and overdue: an audit from a UK primary care medical practice. BMC Fam Pract. 2013;14(170).

15. Hmar RC, Kannangara DRW, Ramasamy SN, Baysari MT, Williams KM, Day RO. Understanding and improving the use of allopurinol in a teaching hospital. Intern Med J. 2015;45(4):383-90.

16. Hughes JC, Wallace JL, Bryant CL, Salvig BE, Fourakre TN, Stone WJ. Monitoring of Urate-Lowering Therapy Among US Veterans Following the 2012 American College of Rheumatology Guidelines for Management of Gout. Ann Pharmacother. 2017;51(4):301-6.

17. Hassan E, Choudry B. The compliance of guidelines set by the British Society for Rheumatology for managing Gout. British Journal of Healthcare Management. 2018;24(1).

18. Jackson G, Dalbeth N, Te Karu L, Winnard D, Gow P, Gerard C, et al. Variation in gout care in Aotearoa New Zealand: a national analysis of quality markers. N Z Med J. 2014;127(1404):37-47.

19. Mikuls TR, Farrar JT, Bilker WB, Fernandes S, Saag KG. Suboptimal physician adherence to quality indicators for the management of gout and asymptomatic hyperuricaemia: results from the UK General Practice Research Database (GPRD). Rheumatology (Oxford). 2005;44(8):1038-42.

20. Clarson LE, Hider SL, Belcher J, Roddy E, Mallen CD. Factors influencing allopurinol initiation in primary care. Ann Fam Med. 2017;15(6):557-60.

21. Kuo C-F, Grainge MJ, Mallen C, Zhang W, Doherty M. Eligibility for and prescription of urate-lowering treatment in patients with incident gout in England. JAMA. 2014;312(24):2684-6.

22. George M, Pullman-Mooar S, Hussain F, Schumacher HR. Evaluating Appropriate Use of Prophylactic Colchicine for Gout Flare Prevention. Arthritis Care Res (Hoboken). 2014;66(8):1258-62.

23. Kerr GS, Richards JS, Nunziato CA, Patterson OV, DuVall SL, Aujero M, et al. Measuring physician adherence with gout quality indicators: a role for natural language processing. Arthritis Care Res (Hoboken). 2015;67(2):273-9.

24. Kapetanovic MC, Hameed M, Turkiewicz A, Neogi T, Saxne T, Jacobsson L, et al. Prevalence and incidence of gout in southern Sweden from the socioeconomic perspective. RMD Open. 2016;2(2):e000326.

25. Meek IL, Vonkeman HE, Van De Laar MAFJ. Hyperuricaemia: A marker of increased cardiovascular risk in rheumatic patients: Analysis of the ACT-CVD cohort. BMC Musculoskelet Disord. 2014;15(1).

26. Alonso A, Rodraguez LA, Logroscino G, Hernan MA. Gout and risk of Parkinson disease: a prospective study. Neurology. 2007;17:1696-16700.

27. Sultan AA, Mallen C, Hayward R, Muller S, Whittle R, Hotston M, et al. Gout and subsequent erectile dysfunction: a population- based cohort study from England. Arthritis Res Ther. 2017;19(1):123.

28. Bevis M, Blagojevic-Bucknall M, Mallen C, Hider S, Roddy E. Comorbidity clusters in people with gout: an observational cohort study with linked medical record review. Rheumatology (Oxford). 2018;57(8):1358-63.

29. Fisher MC, Rai SK, Lu N, Zhang YQ, Choi HK. The unclosing premature mortality gap in gout: a general population-based study. Ann Rheum Dis. 2017;76(7):1289-94.

30. MacFarlane LA, Liu CC, Solomon DH. The effect of initiating pharmacologic insulin on serum uric acid levels in patients with diabetes: A matched cohort analysis. Semin Arthritis Rheum. 2014;44(5):592-6.

31. Olaru L, Soong L, Dhillon S, Yacyshyn E. Coexistent rheumatoid arthritis and gout: a case series and review of the literature. Clin Rheumatol. 2017;36(12):2835-8.

32. Rashid N, Levy GD, Wu YL, Zheng CY, Koblick R, Cheetham TC. Patient and clinical characteristics associated with gout flares in an integrated healthcare system. Rheumatol Int. 2015;35(11):1799-807.

33. Sigurdardottir V, Drivelegka P, Svard A, Jacobsson LTH, Dehlin M. Work disability in gout: a population-based case-control study. Ann Rheum Dis. 2017;77(3):399-404.

34. Chang K, Yokose C, Tenner C, Oh C, Donnino R, Choy-Shan A, et al. Association Between Gout and Aortic Stenosis. Am J Med. 2017;130(2).

35. De Vera M, Rahman MM, Rankin J, Kopec J, Gao X, Choi H. Gout and the risk of Parkinson's disease: a cohort study. Arthritis Rheum. 2008;59(11):1549-54.

36. Landgren AJ, Jacobsson LTH, Lindstrom U, Sandstrom TZS, Drivelegka P, Bjorkman L, et al. Incidence of and risk factors for nephrolithiasis in patients with gout and the general population, a cohort study. Arthritis Res Ther. 2017;19(1).

37. Spaetgens B, de Vries F, Driessen JHM, Leufkens HG, Souverein PC, Boonen A, et al. Risk of infections in patients with gout: a population-based cohort study. Sci Rep. 2017;7.

38. Lu N, Dubreuil M, Zhang Y, Neogi T, Rai SK, Ascherio A, et al. Gout and the risk of Alzheimer's disease: a population-based, BMI-matched cohort study. Ann Rheum Dis. 2015;75(3):547-51.

39. Rho YH, Lu N, Peloquin CE, Man A, Zhu Y, Zhang Y, et al. Independent impact of gout on the risk of diabetes mellitus among women and men: a population-based, BMI-matched cohort study. Ann Rheum Dis. 2014;75(1):91-5.

40. Wahedduddin S, Singh JA, Culhane-Pera KA, Gertner E. Gout in the Hmong in the United States. J Clin Rheumatol. 2010;16(6):262-6.

41. Cheyoe N, Kuning M, Lim A. The prevalence of chronic kidney disease among gout patients in Nongjik hospital, Pattani province. Thai Journal of Pharmaceutical Sciences. 2012;36(4):144-9.

42. Nyberg F, Horne L, Morlock R, Nuevo J, Storgard C, Aiyer L, et al. Comorbidity Burden in Trial-Aligned Patients with Established Gout in Germany, UK, US, and France: A Retrospective Analysis. Advances in Therapy. 2016;33(7):1180-98.

43. Rothenbacher D, Primatesta P, Ferreira A, Cea-Soriano L, Rodriguez LAG. Frequency and risk factors of gout flares in a large population-based cohort of incident gout. Rheumatology (Oxford). 2011;50(5):973-81.

44. Arromdee E, Michet CJ, Crowson CS, O'Fallon WM, Gabriel SE. Epidemiology of gout: is the incidence rising? J Rheumatol. 2002;29(11):2403-6.

45. Lee JH, Yang J, Shin K, Lee GH, Lee WW, Lee EY, et al. Elderly Patients Exhibit Stronger Inflammatory Responses during Gout Attacks. J Korean Med Sci. 2017;32(12):1967-73.

46. Robinson PC, Taylor WJ, Dalbeth N. An Observational Study of Gout Prevalence and Quality of Care in a National Australian General Practice Population. J Rheumatol. 2015;42(9):1702-7.

47. Dehlin M, Drivelegka P, Sigurdardottir V, Svärd A, Jacobsson LTH. Incidence and prevalence of gout in Western Sweden. Arthritis Res Ther. 2016;18(164).

48. Rai SK, Avina-Zubieta JA, McCormick N, De Vera MA, Shojania K, Sayre EC, et al. The rising prevalence and incidence of gout in British Columbia, Canada: Population-based trends from 2000 to 2012. Semin Arthritis Rheum. 2017;46(4):451-6.

49. Kuo C-F, Grainge MJ, Mallen C, Zhang W, Doherty M. Rising burden of gout in the UK but continuing suboptimal management: a nationwide population study. Ann Rheum Dis. 2014;74(4):661-7.

50. Soriano LC, Rothenbacher D, Choi HK, Rodriguez LAG. Contemporary epidemiology of gout in the UK general population. Arthritis Res Ther. 2011;13(2):3.

51. Zarowitz BJ, O'Shea TE. Demographic and clinical profile of nursing facility residents with gout. Consult Pharm. 2013;28(6):370-82.

52. Mikuls TR, Farrar JT, Bilker WB, Fernandes S, Schumacher HR, Jr., Saag KG. Gout epidemiology: results from the UK General Practice Research Database, 1990-1999. Ann Rheum Dis. 2005;64(2):267-72.

53. Maravic M, Hincapie N, Pilet S, Flipo RM, Lioté F. Persistent clinical inertia in gout in 2014: An observational French longitudinal patient database study. Joint Bone Spine. 2018;85(3):311-5.

54. Kuo C-F, Chou I-J, See L-C, Chen J-S, Yu K-H, Luo S-F, et al. Urate-lowering treatment and risk of total joint replacement in patients with gout. Rheumatology (Oxford). 2018;57(12):2129-39.

55. Sultan AA, Whittle R, Muller S, Roddy E, Mallen CD, Bucknall M, et al. Risk of fragility fracture among patients with gout and the effect of urate-lowering therapy. Can Med Assoc J. 2018;190(19):E581-E7.

56. Roughley M, Sultan AA, Clarson L, Muller S, Whittle R, Belcher J, et al. Risk of chronic kidney disease in patients with gout and the impact of urate lowering therapy: A population-based cohort study. Arthritis Res Ther. 2018;20(1).

57. Harrold LR, Mazor KM, Peterson D, Naz N, Firneno C, Yood RA. Patients' knowledge and beliefs concerning gout and its treatment: a population based study. BMC Musculoskelet Disord. 2012;13(180).

58. Dehlin M, Jacobsson LTH. Trends in Gout Hospitalization in Sweden. J Rheumatol. 2018;45(1):145-6.

59. Keenan RT, O'Brien WR, Lee K, Crittenden DB, Fisher MC, Goldfarb DS, et al. Prevalence of contraindications and prescription of pharmacologic therapies for gout. Am J Med. 2011;124(2):155-63.

60. Morlock R, Chevalier P, Horne L, Nuevo J, Storgard C, Aiyer L, et al. Disease Control, Health Resource Use, Healthcare Costs, and Predictors in Gout Patients in the United States, the United Kingdom, Germany, and France: A Retrospective Analysis. Rheumatol Ther. 2016;3(1):53-75.

61. Park JW, Ko DJ, Yoo JJ, Chang SH, Cho HJ, Kang EH, et al. Clinical factors and treatment outcomes associated with failure in the detection of urate crystal in patients with acute gouty arthritis. Korean J Intern Med. 2014;29(3):361-9.

62. Roddy E, Mallen CD, Hider SL, Jordan KP. Prescription and comorbidity screening following consultation for acute gout in primary care. Rheumatology (Oxford). 2010;49(1):105-11.

63. Lin LW, Teng GG, Lim AYN, Yoong JSY. Cost‐effectiveness of an adherence‐enhancing intervention for gout based on real‐world data. Int J Rheum Dis. 2018;16.

64. Janssen CA, Jansen T, Voshaar M, Vonkeman HE, van de Laar M. Quality of care in gout: a clinical audit on treating to the target with urate lowering therapy in real-world gout patients. Rheumatol Int. 2017;37(9):1435-40.

65. Hatoum H, Khanna D, Lin SJ, Akhras KS, Shiozawa A, Khanna P. Achieving Serum Urate Goal: A Comparative Effectiveness Study Between Allopurinol and Febuxostat. Postgrad Med. 2014;126(2):65-75.

66. Jung JY, Choi Y, Suh CH, Yoon D, Kim HA. Effect of fenofibrate on uric acid level in patients with gout. Sci Rep. 2018;8.

67. Pui K, Gow PJ, Dalbeth N. Efficacy and tolerability of probenecid as urate-lowering therapy in gout; clinical experience in high-prevalence population. J Rheumatol. 2013;40(6):872-6.

68. Thueringer JT, Doll NK, Gertner E. Anakinra for the treatment of acute severe gout in critically ill patients. Semin Arthritis Rheum. 2015;45(1):81-5.

69. Kuo CF, Grainge MJ, Mallen C, Zhang WY, Doherty M. Effect of allopurinol on all-cause mortality in adults with incident gout: propensity score-matched landmark analysis. Rheumatology (Oxford). 2015;54(12):2145-50.

70. Dennison EM, Rubin KH, Schwarz P, Harvey NC, Bone KW, Cooper C, et al. Is allopurinol use associated with an excess risk of osteoporotic fracture? A National Prescription Registry study. Arch Osteoporos. 2015;10(36).

71. Solomon DH, Liu CC, Kuo IH, Zak A, Kim SC. Effects of colchicine on risk of cardiovascular events and mortality among patients with gout: a cohort study using electronic medical records linked with Medicare claims. Ann Rheum Dis. 2016;75(9):1674-9.

72. Coburn BW, Michaud K, Bergman DA, Mikuls TR. Allopurinol Dose Escalation and Mortality Among Patients With Gout: A National Propensity-Matched Cohort Study. Arthritis Rheumatol. 2018;70(8):1298-307.

73. Keller SF, Rai SK, Lu N, Oza A, Jorge AM, Zhang Y, et al. Statin use and mortality in gout: A general population-based cohort study. Semin Arthritis Rheum. 2018.

74. Ryu H, Song R, Kim H, Kim J, Lee EY, Lee YJ, et al. Clinical risk factors for adverse events in allopurinol users. J Clin Pharmacol. 2013;53(2):211-6.

75. Crittenden DB, Lehmann RA, Schneck L, Keenan RT, Shah B, Greenberg JD, et al. Colchicine Use Is Associated with Decreased Prevalence of Myocardial Infarction in Patients with Gout. J Rheumatol. 2012;39(7):1458-64.

76. Kwon OC, Hong S, Ghang B, Kim YG, Lee CK, Yoo B. Risk of Colchicine-Associated Myopathy in Gout: Influence of Concomitant Use of Statin. Am J Med. 2016;130(5):583-7.

77. Vargas-Santos AB, Peloquin CE, Zhang YQ, Neogi T. Association of Chronic Kidney Disease With Allopurinol Use in Gout Treatment. JAMA Intern Med. 2018;178(11):1526-33.

78. Lee JS, Won J, Kwon OC, Lee SS, Oh JS. Hepatic Safety of Febuxostat Compared with Allopurinol in Gout Patients with Fatty Liver Disease. J Rheumatol. 2018.

79. Benchimol EI, Smeeth L, Guttmann A, Harron K, Moher D, Petersen I, et al. The REporting of studies Conducted using Observational Routinely-collected health Data (RECORD) Statement. 2015;12(10).
